# Impact of cell culture parameters on production and vascularization bioactivity of mesenchymal stem cell‐derived extracellular vesicles

**DOI:** 10.1002/btm2.10065

**Published:** 2017-06-26

**Authors:** Divya B. Patel, Kelsey M. Gray, Yasasvhinie Santharam, Tek N. Lamichhane, Kimberly M. Stroka, Steven M. Jay

**Affiliations:** ^1^ Fischell Dept. of Bioengineering University of Maryland College Park MD 20742; ^2^ Greenebaum Comprehensive Cancer Center University of Maryland – Baltimore Baltimore MD 21201; ^3^ Biophysics Program University of Maryland College Park MD 20742; ^4^ Center for Stem Cell Biology and Regenerative Medicine University of Maryland – Baltimore Baltimore MD 21201; ^5^ Program in Molecular and Cell Biology University of Maryland College Park MD 20742

**Keywords:** biomanufacturing, EVs, exosomes, mesenchymal stem cells, therapeutic angiogenesis

## Abstract

Mesenchymal stem cell (MSC)‐derived extracellular vesicles (EVs) have emerged as potential therapeutic agents for numerous applications. EVs offer potential advantages over cell‐based therapies with regard to safety, stability and clearance profiles, however production and potency limitations must be addressed to enable eventual translation of EV‐based approaches. Thus, we sought to examine the role of specific cell culture parameters on MSC EV production and bioactivity toward informing rational design parameters for scalable EV biomanufacturing. We report significantly reduced MSC EV vascularization bioactivity, as measured by an endothelial cell gap closure assay, with increasing passage in culture by trypsinization, especially beyond passage 4. We further show that increased frequency of EV collection yielded higher numbers of EVs from the same initial number of MSCs over a 24 hr period. Finally, we demonstrate that decreased cell seeding density in culture flasks resulted in increased production of EVs per cell in MSCs and other cell types. Overall, these studies highlight the need for careful consideration of the parameters of cell passage number and cell seeding density in the production of therapeutic EVs at laboratory scale and for rational design of large‐scale EV biomanufacturing schemes.

## 1 INTRODUCTION

The significance of paracrine mechanisms in mesenchymal stem cell (MSC)‐based therapies for tissue regeneration is well documented.^1^, ^2^, ^3^ Given the extensive use of MSCs in clinical trials, paracrine factors such as chemokines, cytokines, and growth factors have been widely studied,^4^, ^5^, ^6^ whereas extracellular vesicles (EVs) have more recently emerged as important bioactive components of the MSC secretome.^2^, ^7^, ^8^, ^9^ Specifically, MSC‐derived EVs have been shown to reduce myocardial ischemia/reperfusion injury,^10^ ameliorate diabetic nephropathy,^11^ enhance angiogenesis and wound repair,^12^ alleviate liver fibrosis,^13^ and promote osteochondral regeneration,^14^ among many other applications.^15^, ^16^, ^17^, ^18^, ^19^, ^20^, ^21^ Further, the therapeutic application of MSC‐derived EVs offers potential advantages over MSC transplantation via the immunomodulatory properties of EVs as well as from a regulatory perspective due to their expected relative stability and favorable clearance profiles in vivo. EVs could also pose less safety risks than cells in that they cannot divide or differentiate as cells can, likely reducing the potential for tumor formation.^22^ Thus, there is ample justification for increasing interest in clinical application of therapeutic MSC‐derived EVs.

However, barriers to widespread development of MSC EV‐based therapies for human patients remain, including the lack of an established scalable biomanufacturing pathway. Some important progress has been reported, including the generation of immortalized MSC lines for EV production,^9^, ^22^, ^23^ the successful use of MSC EVs in humans,^8^, ^24^ and the development of bioreactors for EV production.^25^ Yet, reports of rational design approaches and/or parameter optimization of EV biomanufacturing are scarce, indicative of inherent inefficiency and lack of knowledge that could hinder clinical translation. For example, MSCs are known to undergo replicative senescence in culture, which impacts their differentiation potential and genetic stability,^26^, ^27^ but the potential connection between this and other cellular events and the cargo and bioactivity of MSC EVs is unknown. Also, MSC cellular bioactivity can be regulated by biophysical stimuli and cell culture configurations,^28^, ^29^, ^30^, ^31^, ^32^, ^33^, ^34^ which might also impact the therapeutic potential of EVs derived from these cells.

Therefore, in these studies, we sought to examine the role of specific cell culture parameters on MSC EV production and bioactivity. We characterized and evaluated EVs isolated from different passages of bone marrow‐derived MSCs to uncover a potential role of genetic instability of MSCs in culture on EV bioactivity. We further assessed the impact of cell seeding density on MSC EVs, hypothesizing that EV production and bioactivity might be regulated based on the totality of the intercellular communication milieu given the well described role of EVs in cell–cell information transfer.^35^, ^36^, ^37^ Finally, we attempted to determine if the findings observed for MSC EVs were conserved in other cell types. Overall, these studies highlight the importance of controlling the cell culture microenvironment for optimal production and potency of therapeutic MSC‐derived EVs.

## 2 RESULTS

### 2.1 MSC EV vascularization bioactivity varies with cell passage

**Figure 1:**
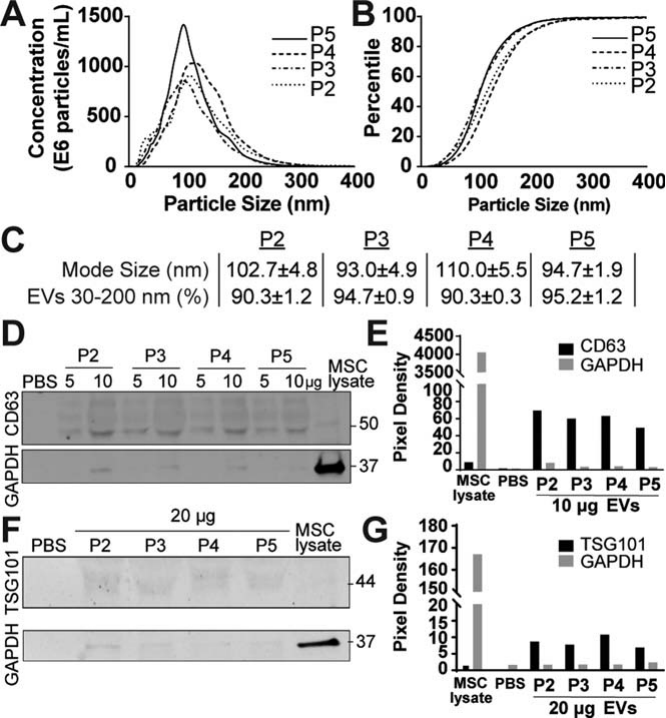
Effect of cell passage on EV characteristics. (A) Concentration and (B) size distribution of Evs isolated from MSCs at different passages (P2–P5) as assessed by NTA. (C) Mode size (diameter) and percentage of EVs measuring between 30 and 200 nm (corresponding to the size range typically defined for exosomes) from MSCs at each passage. Data are representative of three independent experiments (*n* = 3); statistical difference in mode diameter was calculated for P2 versus P5 and P4 versus P5 (*p* < .05); no statistical difference (*p* > .05) was found in EV concentration using one‐way ANOVA with Tukey's multiple comparison test. (D) Immunoblot analysis of exosomal marker CD63 and cellular protein marker GAPDH was conducted for EVs from each MSC passage at 5 and 10 μg of EVs per lane (based on bicinchoninic acid (BCA) analysis of EV surface protein content) and 2.5 μg of MSC lysate (total MSC cellular protein; positive control). PBS was used as a negative control. (E) ImageJ quantification of pixel densities in (D). (F) Immunoblot analysis of exosomal marker TSG101 and cellular protein marker GAPDH was conducted for EVs from each MSC passage at 20 μg of EVs per lane (based on BCA analysis of EV surface protein content) and 2.5 μg of MSC lysate (total MSC cellular protein; positive control). PBS was used as a negative control. (G) ImageJ quantification of pixel densities in (F)

EVs were isolated from conditioned medium of MSCs at various passages (P2, P3, P4, and P5) using differential centrifugation. EVs were then quantified via nanoparticle tracking analysis (NTA) using a NanoSight LM10 (Figure 1a,b). This analysis showed that P2, P3, P4, and P5 MSC‐derived EVs had a peak size of 102.7 ± 4.8, 93.0 ± 4.9, 110.0 ± 5.5, and 94.7 ± 1.9 nm, respectively. Greater than 90% of the total EV population was found to be within a range of 30–200 nm in diameter, regardless of passage number (Figure 1c). Further, immunoblot analysis revealed that CD63 and TSG101, protein markers associated with exosomes,^38^ were enriched uniformly by at least fivefold in the EV samples derived from MSCs at each passage compared to the whole MSC lysate (Figure 1d–g). Taken together, these results suggest a high prevalence of exosomes in the EV populations isolated in these studies.

**Figure 2:**
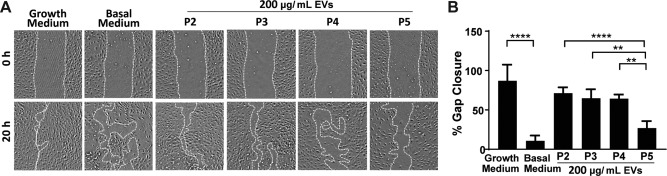
Impact of cell passage on MSC‐derived EV vascularization bioactivity. (A) HDMECs were stimulated with EGM2 medium (growth medium; positive control), EBM2 medium (basal medium; negative control) or 200 μg/ml EVs isolated from MSCs at the indicated passages. Representative images captured at 0 and 20 hr are shown. (B) ImageJ analysis of cell gap area at 20 hr relative to the gap area at 0 hr (gap area depicted with white dotted lines). Data are representative of three independent experiments with three replicates each (*n* = 3). Statistical comparisons determined by two‐way ANOVA with Tukey's multiple comparison tests are shown

The effect of cell passage on the putative pro‐vascularization bioactivity of MSC‐derived EVs was evaluated via an in vitro gap closure assay. All EV populations studied induced an increase in human dermal microvascular endothelial cells (HDMECs) gap closure in response to a 200 µg/ml dose of EVs compared to basal medium (negative control) (Figure 2a). However, a significant decrease in pro‐vascularization bioactivity was observed for EVs isolated from P5 MSCs compared to lower passages (P2: 71.2 ± 7.5%; P3: 64.8 ± 11.3%; P4: 64.2 ± 5.3%; P5: 26.8 ± 8.9%) (Figure 2b).

### 2.2 MSC EV production depends on cell seeding density but not cell passage

**Figure 3:**
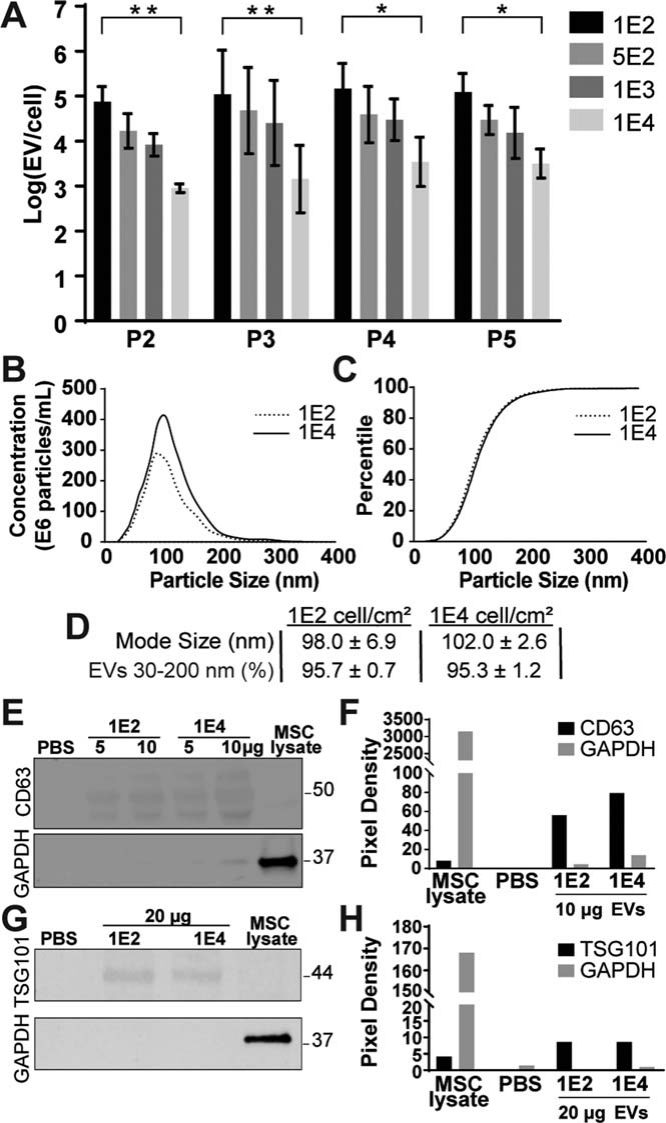
Lower cell seeding density leads to increased EV production rate. (A) MSCs at different passages were seeded at varying initial cell densities (1E2, 5E2, 1E3, and 1E4 cells/cm^2^). Isolated EVs were quantified using NTA and normalized to obtain number of EV produced per cell (EVs/cell). Data are representative of three independent studies (*n* = 3); no significant differences in EV production were calculated between passages. Significant difference was calculated between 1E2 and 1E4 cells/cm^2^ seeding densities for all passages using two‐way ANOVA with Tukey's multiple comparison test (*p < .05, (**p < .01). (B) Concentration and (C) size distribution percentiles of EVs derived from MSCs seeded at 1E2 or 1E4 cells/cm^2^ was determined by NTA. (D) The mode size and EV percentiles for each seeding density EV group are shown. Data are representative of three independent trials (*n* = 3); no significant difference in mode EV diameter was calculated between two densities using unpaired *t* test with Welch's correction. (E) Immunoblot analysis of exosomal marker CD63 and cellular protein marker GAPDH for EVs from each MSC seeding density at 5 and 10 μg (based on BCA analysis of EV surface protein content) of EVs and 2.5 μg of MSC lysate (total MSC cellular protein; positive control). PBS was used as a negative control. (F) ImageJ quantification of pixel densities in (E). (G) Immunoblot analysis of exosomal marker TSG101 and cellular protein marker GAPDH for EVs from each MSC seeding density at 20 μg (based on BCA analysis of EV surface protein content) of EVs and MSC lysate (total MSC cellular protein; positive control). PBS was used as a negative control. (H) ImageJ quantification of pixel densities in (G)

Along with bioactivity, production rate is another critical parameter for potential scalable biomanufacturing of therapeutic EVs. The potential impact of cell passage on MSC EV production was assessed by NTA, with no significant differences observed between passages, regardless of initial cell seeding density (Figure 3). However, seeding density itself was found to be a significant determinant of EV production, as EVs produced per cell decreased between seeding densities of 1E2 cells/cm^2^ and 1E4 cells/cm^2^ for P2, P3, P4, and P5 by ∼100‐fold (*p* < .01), ∼85‐fold (*p* < .01), ∼105‐fold (*p* < .05), and ∼50‐fold (*p* < .05), respectively (*n* = 5) (Figure 3a). The EVs produced by MSCs at these different seeding densities were characterized and found to be similar in size and CD63 and TSG101 expression to each other and to all others in these studies (Figure 3b–g), suggesting that any effect of cell seeding may not be due to a fundamental transformation of the EVs being produced.

**Figure 4:**
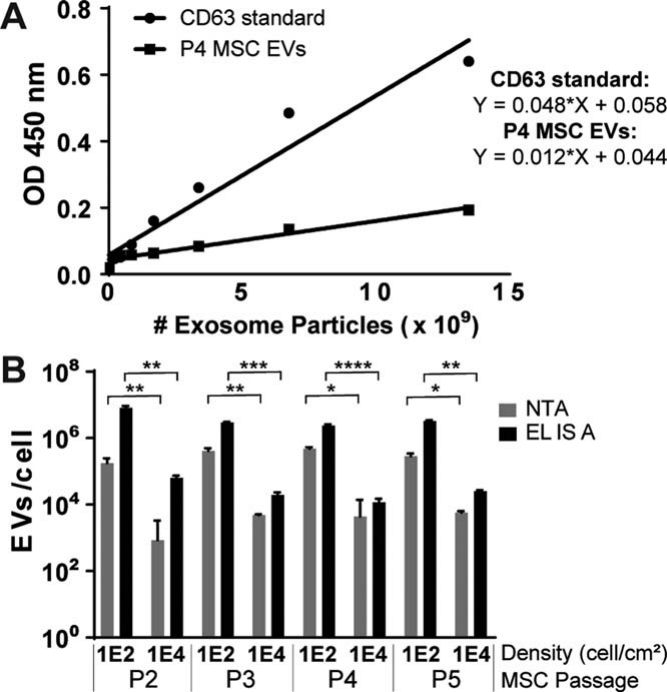
Validation of cell seeding density effects on EV production using CD63‐specific ELISA. (A) Calibration curves for the number of EV particles versus OD450 reading of exosomal CD63 standard or P4 MSCderived EVs. Equations for the line of best fit were determined using linear regression analysis. *R* ^2^ values for CD63 standard and P4 MSC EVs was calculated to be 0.945 and 0.955, respectively. (B) Numbers of EV particles present in isolated EV samples derived from different passage MSCs seeded at 1E2 or 1E4 cells/cm^2^ were determined based on the CD63 standard curve in (A). EV numbers were normalized per cell and the values were compared to NTA data shown in Figure 3a. Data are representative of three independent experiments with three replicates (*n* = 3); statistical significance in EV/cell amount between 1E2 cell/cm^2^ and 1E4 cell/cm^2^ densities was calculated using two‐way ANOVA with Tukey's multiple comparison test (*variance in density EVs for ELISA data; # variance in density EVs for NTA data) (#*p* < .05; ** or ##*p* < .01; ***p < .001; ****p < .0001)

To validate this finding, a CD63 ELISA was conducted to verify EV quantification. Both an exosomal CD63 standard provided by the manufacturer and EVs derived from P4 MSCs were used to create calibration curves for this assay (Figure 4a). Using the equation of the line of best fit derived from a linear regression of the CD63 standard data, EV production from MSCs seeded at different initial densities was quantified. A comparison of ELISA‐based quantification of EV production to NTA‐based quantification from Figure 3a revealed similar trends (Figure 4b). Specifically, we observed decreases in EV production per cell between MSCs seeded at 1E2 or 1E4 cells/cm^2^ for P2, P3, P4, and P5 MSCs measuring ∼126‐fold (*p* < .01), ∼152‐fold (*p* < .001), ∼201‐fold (*p* < .0001), and ∼126‐fold (*p* < .01), respectively (*n* = 3).

### 2.3 MSC EV vascularization bioactivity does not vary with cell density

**Figure 5:**
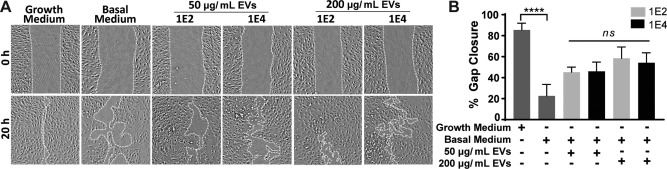
MSC EV vascularization bioactivity does not vary with cell density. (A) HDMECs were stimulated with EGM2 medium (growth medium; positive control), EBM2 medium (basal medium; negative control) or 50 or 200 μg/ml EVs isolated from passage three MSCs seeded at the indicated densities. Representative images captured at 0 and 20 hr are shown. (B) ImageJ analysis of cell gap area at 20 hr relative to the gap area at 0 hr (gap area depicted with white dotted lines). Data represent three independent experiments with three replicates each (*n* = 3); no statistical difference was observed between EVs from MSCs seeded at either density at either concentration using two‐way ANOVA with Tukey's multiple comparison test (ns *p* > .05, ****p < .0001)

Using the same in vitro gap closure assay as in Figure 2, a significant increase in gap closure of HDMECs was observed in the presence of P3 MSC EVs from different seeding densities at both 50 µg/ml (1E2: 45.2 ± 4.8%, *p* < .01; 1E4: 46.1 ± 8.7%, *p* < .01) and 200 µg/ml EV concentration (1E2: 58.4 ± 10.8%, *p* < .05; 1E4: 54.2 ± 9.5%, *p* < .01) compared to untreated HDMECs (basal medium: 22.6 ± 10.9%) (Figure 5a,b). These data were also similar to those observed for P3 MSC EVs in Figure 2. However, two‐way ANOVA with the variance partitioned between seeding density and % gap closure showed no significant difference in gap closure between the two seeding densities (*n* = 3), suggesting that although MSC EV production varies with cell seeding density, there is no significant impact on vascularization bioactivity.

### 2.4 Increased EV collection frequency increases EV production

**Figure 6:**
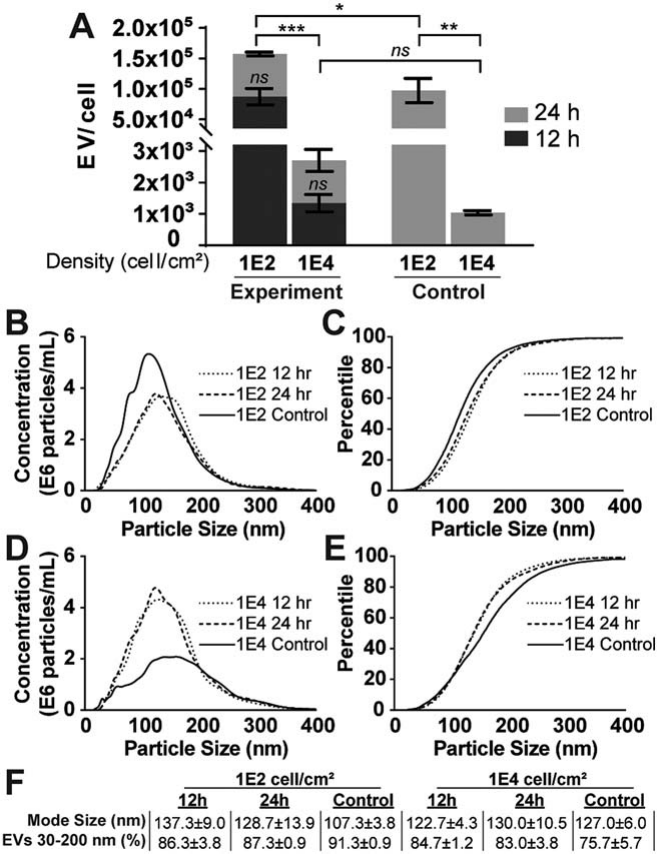
Increased frequency of media collection increases total MSC EV production. (A) NTA quantification of total EVs per cell produced when conditioned media was collected once at 24 hr (control) or twice at 12 and 24 hr (experiment) from MSCs seeded at either 1E2 or 1E4 cells/cm^2^. Data represent three individual experiments (*n* = 3); no significant difference in EV production was calculated between the 12 and 24 hr collection for the “experiment” at each density, nor between the cumulative accumulations for the experimental and control groups at the 1E4 cells/cm^2^ seeding density (ns *p* > .05). Data were analyzed using a two‐way ANOVA with Tukey's multiple comparison analysis (*p < .05, **p < .01, ***p < .001). (B) EV concentration and (C) size distribution was determined by NTA for 1E2 cell/cm^2^ experiment and control. (D) EV concentration and (E) size distribution as determined by NTA are as shown for 1E4 cell/cm^2^ experiment and control. (F) Mode size (diameter) and percentage of EVs measuring between 30 and 200 nm (corresponding to the size range typically defined for exosomes) from MSCs at each cell seeding density. Data are representative of three independent trials (*n* = 3); no statistical difference in mode diameter was calculated for any group using one‐way ANOVA with Tukey's multiple comparison test (*p* > .05)

The finding that cell seeding density impacts MSC EV production combined with the well‐characterized participation of EVs in intercellular information transfer suggests a potential regulatory role of cell–cell communication in EV production rate. One potential manifestation of this regulation would be a change in rate based on the total number of EVs present in the system. Thus, the effects of medium collection and replacement frequency on EV production by P3 MSCs were assessed. MSCs were seeded at 1E2 or 1E4 cells/cm^2^ and medium was collected once (at 24 hr) or twice (at 12 and 24 hr) over the same total time period. Quantification of EVs via NTA revealed that total EV production increased ∼1.6‐fold (1E2 cells/cm^2^, *p* < .05) and ∼2.6‐fold (1E4 cells/cm^2^, *p* > .05) over 24 hr when medium was collected at 12 hr in addition to the 24 hr collection (Figure 6a). Moreover, collection frequency of conditioned medium from MSCs at a 1E2 cells/cm^2^ density every 3 hr for 6 hr total, or every 6 hr for 12 hr total led to 2.0‐ and 2.4‐fold increases, respectively, compared to a single collection at the latter time point (Supporting Information Figure S1). Collection frequency did not significantly impact EV structure, as the peak sizes all remained within the exosomal range (Figure 6b,c and Supporting Information Figure S1).

### 2.5 Variance of EV production with cell seeding density is not limited to MSCs

**Figure 7:**
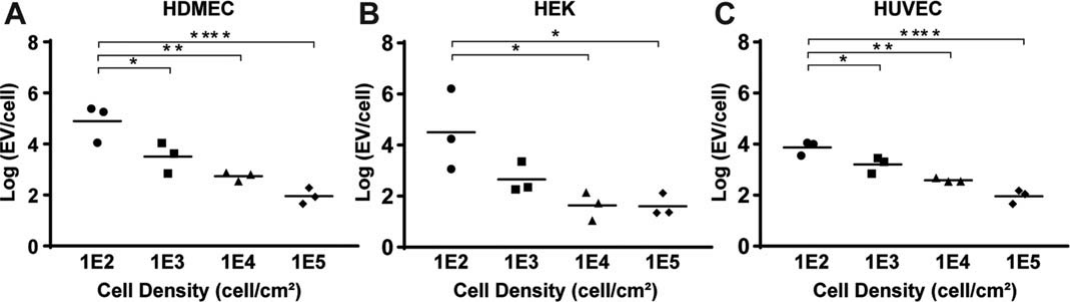
Variance of EV production with cell seeding density is conserved across multiple cell types. NTA quantification of EVs per cell produced by (A) HDMECs, (B) HEK cells, and (C) HUVECs seeded at 1E2, 1E3, 1E4, and 1E5 cells/cm^2^. Data are representative of three individual experiments (*n* = 3); significant differences in EV production at each seeding density were calculated using one‐way ANOVA with Tukey's multiple comparison test (*p < .05; **p < .01; ****p < .0001)

The effects of cell passage on MSCs are well characterized and not likely to be generalized across cell types. However, it was unclear if the observed cell seeding density effects on EV production were specific to MSCs. Thus, this effect was investigated in other EV‐producing cell types that might have relevant therapeutic applications. EVs were collected (single collection at 24 hr) from HDMECs,^39^ human embryonic kidney 293T (HEK) cells,^40^, ^41^ or human umbilical vein endothelial cells (HUVECs)^40^, ^42^ seeded at various densities (1E2, 1E3, 1E4, or 1E5 cells/cm^2^). Quantification by NTA revealed significant differences between seeding densities in all cell types, with the general trend of increased EV production at lower cell seeding densities (Figure 7). These results are consistent with those observed for MSCs (Figure 3) and suggest that cell density may be a general determinant of EV production and thus should be carefully considered and controlled when developing a scalable biomanufacturing approach for therapeutic EVs.

## 3 DISCUSSION

Putatively, MSC proliferation, differentiation, and bioactivity can significantly vary based on the cell culture microenvironment.^28^, ^29^, ^30^ Parameters such as time in culture, shear stress, oxygen content, medium composition, and cell–material interactions have been shown to impact MSCs.^28^, ^29^, ^30^, ^31^, ^32^, ^33^, ^34^ Furthermore, parameters such as 2‐dimensional (2D) versus 3‐dimensional (3D) culture^43^ as well as static versus dynamic culture^25^ have been reported to alter EV production and bioactivity. Here, we aimed to investigate how some of the cellular changes induced by various culture parameters might affect the production and bioactivity of EVs from MSCs in 2D static culture (which remains commonly used). Our results suggest that although the production rate and size distribution of EVs do not change with increasing MSC passage in culture (Figure 1), the vascularization bioactivity of the EVs declines significantly (Figure 2). Increased MSC passage is associated with alterations in genes involving cell cycle, protein ubiquitination, and apoptosis,^27^ all of which can result in decreased cellular activity.^26^, ^27^ Our data suggest that this diminished activity also impacts EV function, indicative that it is essential to maintain MSCs in a non‐senescent state to retain the therapeutic potential of their EVs.

The results of this study also indicate that seeding MSCs at low initial densities leads to higher levels of EV production (Figures 3 and 4). This could be due to metabolic effects, and these data may also reflect previous studies that have linked lower cell seeding densities with more rapid proliferation of MSCs as well as higher percentages of multipotent cells.^44^, ^45^ Additionally, reduced cell–cell contacts at low seeding densities may also play a role in the observed increase in EV production, as EV generation may be a compensatory intercellular communication mechanism. This idea is supported by the data in Figure 6 and Supporting Information Figure S1, which show that removing EVs from the culture microenvironment more frequently results in increased total EV production. Further, lower levels of cell–cell contacts may induce an increase in total available cell membrane surface area to allow increased budding of microvesicles, a subtype of EVs.^46^ EV production could also be limited at higher cell densities by contact inhibition effects.^47^ Interestingly, cell density effects on EV production were observed in multiple cell lines associated with vascularization (Figure 7), suggesting a conserved mechanism.

While decreased cell seeding density led to increased EV production, we found that EV vascularization bioactivity was independent of this parameter (Figure 5). This result defied our expectation of reduced EV bioactivity from more densely packed cells, which was based on previous reports that contact inhibition reduces secretion of growth factors of MSCs.^26^, ^44^ In total, our data support the conclusion that cellular changes incurred through passaging are more critical in defining the bioactivity of MSC EVs than seeding density of the EV‐producing cells. Further study is required to identify the mechanistic links between these cellular changes and alteration of EV‐associated lipids, proteins, and/or nucleic acid cargo.

Overall, these data point to several biological phenomena of which better understanding could lead to rational design of MSC EV biomanufacturing for therapeutic applications. EV production by MSCs and other cells may be responsive to the requirements of EVs for intercellular communication in the cell culture microenvironment. Thus, further understanding of the regulatory roles of EVs in cell–cell communication could define design parameters for a therapeutic EV biomanufacturing platform. Additionally, clarification of specific components that impart bioactivity to MSC EVs will be crucial. Many studies have implicated specific microRNAs (miRNAs) as key players,^35^, ^37^, ^48^ however, these data must be balanced against the apparent energetic inefficiency of EV‐mediated miRNA transfer suggested by the low levels of miRNA found per EV in general.^49^ Beyond this clarification, further understanding of the mechanisms linking cellular changes incurred during passaging of MSCs in conventional cell culture systems and the corresponding changes in critical EV components would further inform rational design of therapeutic MSC EV production for applications in therapeutic vascularization and beyond. These data also suggest that further optimization of MSC EV production may be achieved through additional modulation of cell density. However, the potential benefit of this from an economic standpoint would have to be weighed with better understanding of how EV production on a per cell basis would impact total production costs. Future studies focused on parsing the roles of other cell culture parameters such as dimensionality and dynamic culturing on EV production may be required to engineer a complete culture microenvironment for enhanced EV production. Nevertheless, it seems clear at least that researchers working with MSC EVs should carefully control MSC density and passage number to maximize reproducibility and efficiency.

## 4 CONCLUSIONS

The data reported here suggest that production of MSC EVs for therapeutic vascularization applications can be controllably enhanced by seeding cells at low densities and using only low passages for EV collection. Further, more frequent collection of EVs enables enhanced total production, suggesting that continuous culture systems may improve EV yield. The increase in EV production per cell at lower seeding densities was observed across multiple cell types, thus these findings have broader implications beyond bone marrow‐derived MSCs and should be considered by all researchers and companies developing EV‐based therapies and/or EV biomanufacturing approaches.

## 5 MATERIALS AND METHODS

### 5.1 Cell culture

Bone marrow‐derived MSCs and HEK 293T cells were obtained from ATCC (MSC: PCS‐500–012; HEK: CRL‐3216). HDMECs were obtained from PromoCell (C‐12212) and HUVECs were obtained from Lonza (C2519A). Tissue culture polystyrene flasks were coated with 0.1% gelatin at 37°C for 1 hr prior to seeding HDMECs and HUVECs. All cell types were cultured in the designated growth culture medium in the presence of EV‐depleted serum as previously described.^50^ Cell passage for MSCs was designated as P1 upon arrival from the manufacturer.

### 5.2 EV collection

For MSC passage experiments, cells were seeded into tissue culture flasks at their respective experimental passage (P2–P5). Upon reaching approximately 80% confluence, the cell medium was changed to EV‐depleted medium for 24 hr, after which the medium was collected and termed “conditioned medium” until EV isolation.

For density experiments, cells were seeded at their respective densities (i.e., 1E2, 5E2, 1E3, 1E4, or 1E5 cells/cm^2^) into T‐25 tissue culture flasks. After 24 hr, the medium was changed to 5 ml of the respective EV‐depleted medium. After 24 hr culture in EV‐depleted medium, conditioned medium was collected for EV isolation. Flasks were subsequently trypsinized and cells were counted to ultimately attain an EV/cell measure.

For collection frequency experiments, MSCs were seeded into four T‐25 flasks, two of each 1E4 and 1E2 cells/cm^2^ density. After 12 hr, cell medium was changed to the EV‐depleted formulation (*t* = 0 hr). Experimental groups consisted of one flask from each density, and control groups consisted of the other flask from each density. Media from the experimental groups were collected after 12 hr of EV‐depleted medium culture (*t* = 12 hr) and replaced with fresh EV‐depleted medium. Media were collected from all groups 24 hr after the initial addition of EV‐depleted medium (*t* = 24 hr) for EV isolation. All flasks were trypsinized and cells were counted to ultimately attain an EV/cell measure. EV/cell was estimated using the 24 hr counts for both 12 and 24 hr time points. Similar studies were performed with increased collection frequencies as detailed in the supplemental data.

### 5.3 EV isolation

Conditioned media from all cells at appropriate seeding densities and passages were collected after 24 hr of culture unless otherwise stated. EVs were isolated by differential centrifugation with 100,000 × *g* as the final centrifugation step as previously described.^50^ Pelleted EVs were resuspended in 1X PBS and subsequently washed with 1X PBS using Nanosep 300 kDa MWCO spin columns (OD300C35; Pall). EVs were resuspended again in 1X PBS and total protein was measured by BCA assay. The average total protein from 25 ml of conditioned medium ranged from 100 to 200 µg.

### 5.4 EV quantification by NTA

EVs were diluted to a concentration of 1–10 µg of protein/ml to achieve 20–100 objects per frame. Samples were manually injected into the sample chamber at ambient temperature. Each sample was measured in triplicate at camera setting 14 with an acquisition time of 30 s and detection threshold setting of 7. At least 200 completed tracks were analyzed per video. NTA analytical software version 2.3 was used for capturing and analyzing the data.

### 5.5 EV quantification by CD63 ELISA

The concentration of EVs was determined by the amount of total immunoreactive EV‐associated CD63 (ExoELISA™, System Biosciences, Mountain View, CA). Briefly, 5 or 10 μg of EVs (by protein mass) were immobilized in 96‐well microtiter plates and incubated overnight at 37°C (binding step). Plates were washed three times for 5 min using a wash buffer solution and then incubated with primary antibody (CD63) at room temperature (RT) for 1 hr under agitation. Plates were washed and incubated with secondary antibody (1:5000) at RT 1 hr under agitation. Plates were washed and incubated with super‐sensitive TMB ELISA substrate at RT for 45 min under agitation. The reaction was terminated using Stop Buffer solution. Absorbance was measured at 450 nm. The number of EVs/ml was obtained using an exosomal CD63 standard curve calibrated against NTA data (number of EVs). Final data was expressed as the number of EVs/cell for each respective data set.

### 5.6 Immunoblots

The levels of CD63, TSG101, and GAPDH, were quantified by immunoblot analysis as described previously^50^ using antibodies against CD63 (H‐193; Santa Cruz, sc‐15363) at 1:200, TSG101 (C‐2; Santa Cruz, sc‐7964) at 1:200 and GAPDH (D16H11; Cell Signaling, 5174) at 1:2000. Goat anti‐rabbit IRDye 800CW (925–32210; LICOR) and Goat anti‐mouse IRDye 680RD (925–68070; LICOR) secondary antibodies were used at a dilution of 1:10,000. Bands were detected with a LI‐COR Odyssey CLX Imager and the data were quantified using ImageJ.

### 5.7 Gap closure assay

HDMECs were seeded in 48‐well plates at 40,000 cells/well in endothelial cell growth medium (EGM2; Lonza, CC‐3162) and allowed to grow until formation of a uniform monolayer. The cell monolayer was disrupted using a pipette tip and the medium was replaced with endothelial cell basal medium (EBM2; Lonza, CC‐3156), with or without the addition EVs at 50 or 200 µg/ml. EBM2 or EGM2 were added for negative or positive control, respectively. After 20 hr the closure of the cell gap was determined using ImageJ. To determine gap closure, all gaps between cells at 20 hr larger than gaps between cells in the monolayer at 0 hr were traced and summed together to calculate the total gap area at 20 hr. This value was taken relative to the gap area at 0 hr, converted to a percent, and subtracted from 100% to quantify % gap closure.

### 5.8 Statistics

Data are presented as mean ± SEM. Differences between groups were analyzed by Student's *t* test for two samples. One‐way ANOVA was used for comparing data sets of three or more, while a two‐way ANOVA was used for grouped data sets. Tukey's multiple comparison test was used to determine significant differences (*p* < .05). Notation for significance in figures are as follows: ns *p* > .05, ^#^or *p < .05; ^##^or **p < .01; ***p < .001; ****p < .0001.

## CONFLICT OF INTEREST

The authors declare no conflicts of interest.

## Supporting information

Additional Supporting Information may be found online in the supporting information tab for this article.

**FIGURE S1** Effect of short interval medium collection frequency on total MSC EV production. (A) NTA quantification of total EVs per cell produced when conditioned medium was collected at 6 hr only (control) compared to collections at 3 and 6 hr (experiment) from MSCs seeded at 1E2 cells/cm^2^. Data represent 3 individual experiments (n=3); no significant difference in EV production was calculated between the 3 h and 6 h collection times for the “experiment” or between the cumulative sum collected from the “experiment” versus “control” groups (ns *P*>0.05). Data were analyzed using a two‐way ANOVA with Tukey's multiple comparison analysis. (B) NTA quantification of total EVs per cell produced when conditioned medium was collected at 12 h only (control) or at 6 h and 12 h (experiment) from MSCs seeded at 1E2 cells/cm^2^. Data represent 3 individual experiments (n=3); no significant difference in EV production was calculated between the 6 h and 12 h collection times for the “experiment” or between the cumulative sum collected from the “experiment” versus the “control” groups (ns *P*>0.05). Data were analyzed using a two‐way ANOVA with Tukey's multiple comparison analysis. (C) EV concentration and (D) size distribution were determined by NTA for the experiment and control groups from the 6 h study. (E) EV concentration and (F) size distribution as determined by NTA are as shown for the experiment and control groups of the 12 h study. (G) Mode size (diameter) and percentage of EVs measuring between 30 and 200 nm (corresponding to the size range typically defined for exosomes) from MSCs in each study. Data are representative of 3 independent trials (n=3); no statistical difference in mode diameter was calculated for any group using one‐way ANOVA with Tukey's multiple comparison test (*P*>0.05)
