# Supplementary material for: Impact of cell culture parameters on production and vascularization bioactivity of mesenchymal stem cell‐derived extracellular vesicles
Source: Bioeng Transl Med. 2017 Jun 26;2(2):170–9. doi: 10.1002/btm2.10065 (PMC5579732; doi:10.1002/btm2.10065)
Supplement: Supplementary file 1 — FIGURE S1 Effect of short interval medium collection frequency on total MSC EV production. (A) NTA quantification of total EVs per cell produced when conditioned medium was collected at 6 hr only (control) compared to collections at 3 and 6 hr (experiment) from MSCs seeded at 1E2 cells/cm2. Data represent 3 individual experiments (n=3); no significant difference in EV production was calculated between the 3 h and 6 h collection times for the “experiment” or between the cumulative sum collected from the “experiment” versus “control” groups (ns P>0.05). Data were analyzed using a two‐way ANOVA with Tukey's multiple comparison analysis. (B) NTA quantification of total EVs per cell produced when conditioned medium was collected at 12 h only (control) or at 6 h and 12 h (experiment) from MSCs seeded at 1E2 cells/cm2. Data represent 3 individual experiments (n=3); no significant difference in EV production was calculated between the 6 h and 12 h collection times for the “experiment” or between the cumulative sum collected from the “experiment” versus the “control” groups (ns P>0.05). Data were analyzed using a two‐way ANOVA with Tukey's multiple comparison analysis. (C) EV concentration and (D) size distribution were determined by NTA for the experiment and control groups from the 6 h study. (E) EV concentration and (F) size distribution as determined by NTA are as shown for the experiment and control groups of the 12 h study. (G) Mode size (diameter) and percentage of EVs measuring between 30 and 200 nm (corresponding to the size range typically defined for exosomes) from MSCs in each study. Data are representative of 3 independent trials (n=3); no statistical difference in mode diameter was calculated for any group using one‐way ANOVA with Tukey's multiple comparison test (P>0.05) [file BTM2-2-170-s001.pdf]

SUPPORTING INFORMATION

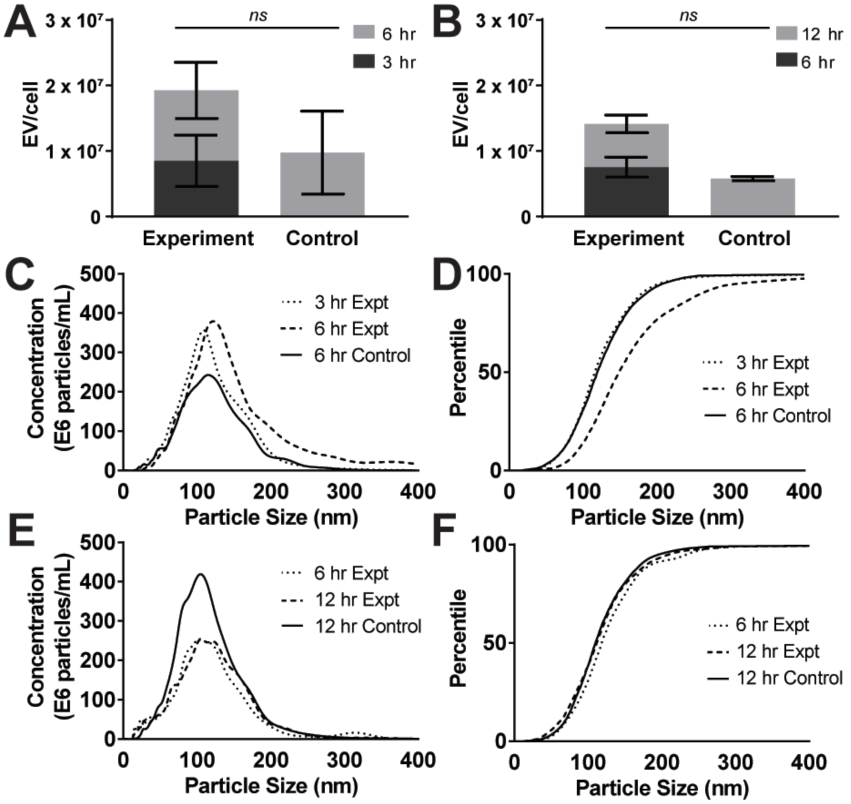

G

	6 hr Study			12 hr Study		
	**3h**	**6h**	**Control**	**6h**	**12h**	**Control**
**Mode Size (nm)**	107.7±1.5	130.7±12.6	112.7±5.5	113.3±6.7	117.7±7.4	105.3±2.7
**EVs 30-200 nm (%)**	93.3±0.7	76.3±13.7	91.7±3.0	91.3±2.7	92.0±0.6	94.0±0.6

Figure S1: Effect of short interval medium collection frequency on total MSC EV production. (A) NTA quantification of total EVs per cell produced when conditioned medium was collected at 6 h only (control) compared to collections at 3 h and 6 h (experiment) from MSCs seeded at 1E2 cells/cm^2. Data represent 3 individual experiments (n=3); no significant difference in EV production was calculated between the 3 h and 6 h collection times for the "experiment" or between the cumulative sum collected from the "experiment" versus "control" groups (ns *P*>0.05). Data were analyzed using a two-way ANOVA with Tukey's multiple comparison analysis. (B) NTA quantification of total EVs per cell produced when conditioned medium was collected at 12 h only (control) or at 6 h and 12 h (experiment) from MSCs seeded at 1E2 cells/cm^2. Data represent 3 individual experiments (n=3); no significant difference in EV production was calculated between the 6 h and 12 h collection times for the "experiment" or between the cumulative sum collected from the "experiment" versus the "control" groups (ns *P*>0.05). Data were analyzed using a two-way ANOVA with Tukey's multiple comparison analysis. (C) EV concentration and (D) size distribution were determined by NTA for the experiment and control groups from the 6 h study. (E) EV concentration and (F) size distribution as determined by NTA are as shown for the experiment and control groups of the 12 h study. (G) Mode size (diameter) and percentage of EVs measuring between 30-200 nm (corresponding to the size range typically defined for exosomes) from MSCs in each study. Data are representative of 3 independent trials (n=3); no statistical difference in mode diameter was calculated for any group using one-way ANOVA with Tukey's multiple comparison test (*P*>0.05).
